# Nationwide improvements in geriatric mortality due to traumatic brain injury in Japan

**DOI:** 10.1186/s12873-022-00577-w

**Published:** 2022-02-10

**Authors:** Sanae Hosomi, Tomotaka Sobue, Tetsuhisa Kitamura, Hiroshi Ogura, Takeshi Shimazu

**Affiliations:** 1 Department of Traumatology and Acute Critical Medicine, Osaka University Graduate School of Medicine, 2-15 Yamadaoka, Suita-shi, Osaka, 565-0871 Japan; 2 Division of Environmental Medicine and Population Sciences, Department of Social and Environmental Medicine, Osaka University Graduate School of Medicine, 2-2, Yamada-oka, Suita, Japan

**Keywords:** Aging, Mortality, Traumatic brain injury

## Abstract

**Background:**

Traumatic brain injury (TBI), both isolated and in combination with extracranial lesions, is a global health problem associated with high mortality. Among various risk factors for poor clinical outcomes, age is the most important independent predictor of mortality in patients with TBI. TBI-related mortality is expected to increase as the society ages. However, in a super-aged society such as Japan, little is known about the trend of TBI-related mortality among older adults. Herein, we assessed the nationwide trend of the incidence and clinical outcomes of geriatric patients with TBI in Japan using the national Japanese Trauma Data Bank (JTDB) registry.

**Methods:**

In this retrospective cohort study, cases of TBI (aged ≥65 years) in hospitals registered with the JTDB database between January 2004 and December 2018 were included. In-hospital mortality was the primary outcome, and mortality in the emergency department was the secondary outcome. The odds ratios (ORs) and 95% confidence intervals (CIs) for in-hospital deaths with respect to 3-year periods were assessed using multivariable analysis after adjusting for potential confounders.

**Results:**

The main cause of TBI in older individuals was falls. The proportion of patients who died after hospitalization during the study period decreased markedly from 29.5% (194/657) during 2004–2006 to 14.2% (1309/9240) during 2016–2018 in the isolated TBI group (adjusted OR = 0.42, 95% CI: 0.33–0.53) and from 48.0% (119/248) during 2004–2006 to 21.7% (689/3172) during 2016–2018 in the multiple trauma group (adjusted OR = 0.32, 95% CI: 0.23–0.45). The adjusted ORs for the 3-year increment were 0.84 (95% CI: 0.81–0.88) and 0.78 (95% CI: 0.75–0.83) for the isolated TBI and multiple trauma groups, respectively.

**Conclusions:**

Using the national JTDB registry, we demonstrated a nationwide reduction in TBI-related mortality. Our findings in the super-aged society of Japan may provide insight for the treatment of geriatric patients with TBI worldwide.

**Supplementary Information:**

The online version contains supplementary material available at 10.1186/s12873-022-00577-w.

## Background

Both isolated traumatic brain injury (TBI) and its combination with extracranial lesions are global health problems associated with high mortality, and their treatment is expensive [1–3]. While the incidence and causes of TBI vary across countries, TBI is a major cause of mortality in young people and in people aged ≥65 years, especially those in developed countries [4, 5]. TBI-related mortality is affected by many factors, including extracranial injuries and preexisting medical conditions. Among various factors affecting outcome, age is an important independent predictor of mortality in patients with TBI [6].

The aged population is rapidly growing worldwide. According to the World Health Organization, the proportion of a society’s population comprising persons aged ≥65 years influences its “aging rate.” A society with an aging rate of 21% is considered a “super-aged society.” In 2019, Japan had an aging rate of 28.4%; this is the highest aging rate worldwide and is unprecedented in absolute terms. In an aging society, TBI-related mortality has been reported to be worse in older adults than that in other age groups; however, this was based on data obtained from relatively small geographic areas [7, 8]. There is limited evidence on nationwide trends of TBI-related mortality among older adults in a super-aged society.

The Japanese Trauma Data Bank (JTDB) was launched in 2003 by the Japanese Association for the Surgery of Trauma (Trauma Surgery Committee) and the Japanese Association for Acute Medicine (Committee for Clinical Care Evaluation) [9]; it is similar to trauma databases in North America, Europe, and Oceania. The JTDB records the data of patients with trauma, including age; sex; cause of injury; AIS code (version 1998); ISS; vital signs at hospital arrival; date and time series from hospital arrival to discharge; clinical, radiological, or surgical interventions; complications; in-hospital death; and the probability of survival calculated using the Trauma Injury Severity Score (TRISS) [9]. By 2018, 272 major emergency medical institutions across Japan had been registered in the JTDB database [9]. The included hospitals have service levels similar to those of Level I trauma centers in the USA [10].

Here, we aimed to assess nationwide trends in the incidence and outcomes of geriatric patients with TBI in Japan using data from the national JTDB registry. We assessed trends in elderly patients with TBI. Furthermore, we considered isolated TBI and multiple trauma (a combination of trauma in the head and other regions) separately as trauma in multiple regions has been associated with a greater risk of traumatic death compared with isolated TBI [11]. Since the increasing number of elderly patients affects total mortality, we hypothesized that the trend of TBI-related mortality would worsen as society ages.

## Methods

### Study design, population, and setting

This study was approved by the ethics committee of Osaka University Graduate School of Medicine (No. 16260). In this retrospective cohort study, cases of TBI in hospitals registered with the JTDB database between January 2004 and December 2018 were included. TBI was defined as any injury to the internal contents of the skull, including the brainstem, cerebellum, and cerebrum, with an Abbreviated Injury Scale (AIS) code [12]. AIS is an anatomically based injury severity scoring system that classifies various types of trauma based on their anatomical location and severity on a 6-point scale, and the AIS score is reported to correspond well with the Marshall CT score [13]. Patients with other critical injuries (AIS ≥ 3) due to multiple trauma were separated from those with an isolated TBI [14]. Patients who met the following criteria were excluded: had an AIS code of 6 (nonsurvivable injury) or AIS code of 9 (unspecified injury); required inter-hospital transport; had cardiac arrest at hospital arrival [15, 16]; or had missing data for age, sex, Glasgow Coma Scale score (GCS) on hospital arrival, Injury Severity Score (ISS), or survival outcome.

In this study, patients in cardiac arrest were defined as those whose systolic blood pressure was 0 mmHg and/or heart rate was 0 bpm at the time of hospital arrival [16].

### Japanese trauma data Bank

From all participating hospitals, data were collected through the Internet. In all these hospitals, the data were mainly entered by physicians and medical assistants who had attended an AIS-coding course [9, 16]. The data used in this study are the most recent data available in this registry.

### Study endpoints

The primary outcome of this investigation was in-hospital death, and the secondary outcome was mortality in the emergency department (ED).

### Statistical analysis

Descriptive data are expressed as counts and percentages for categorical variables and as median with interquartile range for numerical variables. The annual trends in baseline characteristics with respect to 3-year periods (2004–2006, 2007–2009, 2010–2012, 2013–2015, 2016–2018] were assessed using linear trend tests. To assess the improvement in mortality over time, the outcomes with respect to 3-year periods were evaluated using univariable and multivariable logistic regression analyses. Based on these analyses, we calculated the odds ratio (OR) and 95% confidence interval (CI) for the variables. The clinically relevant confounding variables were selected from previous reports and adjusted for the analyses [17–21]. For the multivariable logistic regression analyses, the following 12 variables were adjusted for isolated TBI: age (65–69, 70–74, 75–79, 80–84, 85–89, 90–94, 95–99, and ≥ 100), sex (male, female), type of injury (blunt; no, yes), mechanism of trauma (traffic accident, fall, others), cause of trauma (accident; no, yes), transfer system (ambulance, physician staffed ambulance/helicopter, others), GCS category on arrival (severe, GCS score of 3–8; moderate, GCS score of 9–12; mild, GCS score of 13–15), hypotension (systolic blood pressure ≤ 90 mmHg) on admission to the ED (no, yes), an indication of surgery for TBI (no, yes), use of anticoagulant or antiplatelet drugs (no, yes), major comorbidities (hypertension, diabetes mellitus, chronic obstructive pulmonary disease, and so on) (no, yes), and maximum head AIS scores of 3,4, and 5. In the multivariable logistic regression model for multiple trauma, ISS (continuous value), an indication of surgical intervention for other region injury (no, yes), and the 12 aforementioned variables (isolated TBI) were adjusted.

Statistical significance was defined as two-sided *p*-values < 0.05 for the trend of patient characteristics, and 95% CI was calculated for the trend of mortality. Analyses were performed using STATA, version 16 (StataCorp, College Station, TX, USA).

This manuscript was drafted in accordance to the STROBE statement for comprehensive reporting of cohort and cross-sectional studies [22].

## Results

**Fig. 1:**
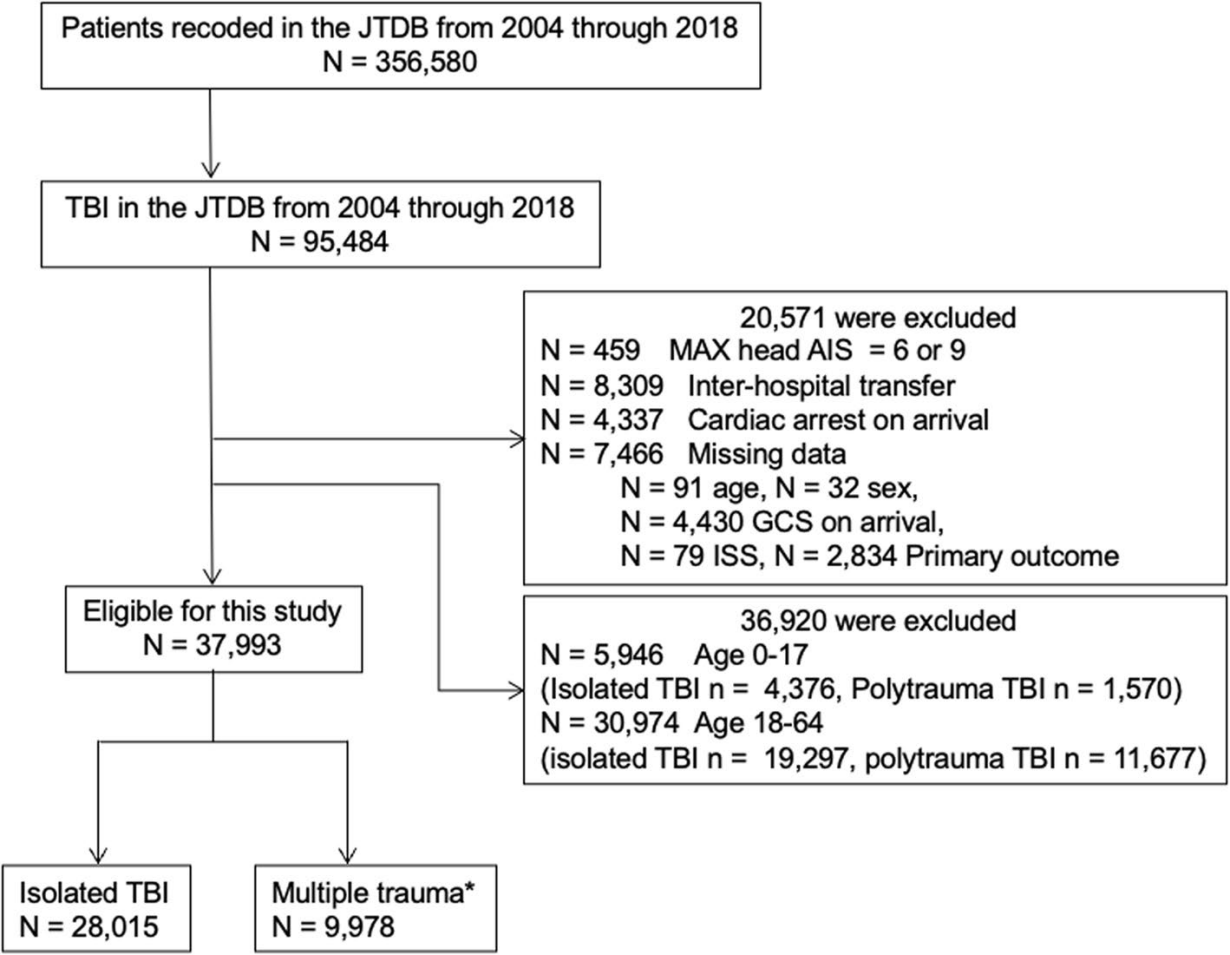
Flowchart of the selection of patients included in this study. *Multiple trauma: TBI and AIS ≥3 for injury in other areas. TBI = traumatic brain injury, JTDB = Japan Trauma Data Bank, AIS = Abbreviated Injury Scale, GCS = Glasgow Coma Scale

In this study, a total of 37,993 patients were included. Of these patients, 28,015 (73.74%) had isolated TBI and 9978 (26.26%) had multiple trauma (Fig. 1).

Baseline characteristics of patients with isolated TBI and multiple trauma are presented in Table 1 and Table 2, respectively. Median ages for the isolated TBI and multiple trauma groups were 78 (72–83) years and 76 (70–82) years, respectively. In each 3-year interval during the study period, the median age of the patients increased. In both the isolated TBI and multiple trauma groups, the proportion of patients in the 65–74-year age group tended to decrease over time, while that in the ≥85-year age group tended to increase. The proportion of men was similar in the isolated TBI and multiple trauma groups throughout the study.

**Table 1:** Baseline characteristics of isolated TBI patients by year

		Total	2004–2006	2007–2009	2010–2012	2013–2015	2016–2018	p for trend
		*N* = 28,015	*N* = 657	*N* = 2668	*N* = 5878	*N* = 9572	*N* = 9240	
Age	Median (IQR), years	78 (72–83)	74 (69–80)	76 (70–81)	77 (71–83)	78 (72–84)	79 (72–84)	< 0.001
	65–74	10,294 (36.7%)	331 (50.4%)	1154 (43.3%)	2270 (38.6%)	3455 (36.1%)	3084 (33.4%)	< 0.001
	75–84	11,753 (42.0%)	250 (38.1%)	1089 (40.8%)	2498 (42.5%)	4059 (42.4%)	3857 (41.7%)	0.323
	85-	5968 (21.3%)	76 (11.6%)	425 (15.9%)	1110 (18.9%)	2058 (21.5%)	2299 (24.9%)	< 0.001
Sex	Male, n(%)	17,124 (61.1%)	399 (60.7%)	1639 (61.4%)	3595 (61.2%)	5880 (61.4%)	5611 (60.7%)	0.580
Type of trauma	Blunt, n(%)	27,111 (96.8%)	625 (95.1%)	2563 (96.1%)	5638 (95.9%)	9290 (97.1%)	8995 (97.3%)	< 0.001
Mechanism of trauma	Traffic accident, n(%)	7107 (25.4%)	242 (36.8%)	899 (33.7%)	1581 (26.9%)	2325 (24.3%)	2060 (22.3%)	< 0.001
	Fall, n(%)	18,582 (66.3%)	336 (51.1%)	1528 (57.3%)	3782 (64.3%)	6497 (67.9%)	6439 (69.7%)	< 0.001
	Others, n(%)	2326 (8.3%)	79 (12.0%)	241 (9.0%)	515 (8.8%)	750 (7.8%)	741 (8.0%)	0.001
Cause of trauma	Accident, n(%)	25,497 (91.0%)	592 (90.1%)	2379 (89.2%)	5325 (90.6%)	8761 (91.5%)	8440 (91.3%)	0.001
Transfer system	Ambulance, n(%)	23,283 (83.1%)	570 (86.8%)	2280 (85.5%)	4933 (83.9%)	7898 (82.5%)	7602 (82.3%)	< 0.001
	Physician staffed ambulance/helicopter, n(%)	2191 (7.8%)	35 (5.3%)	161 (6.0%)	398 (6.8%)	792 (8.3%)	805 (8.7%)	< 0.001
	Others, n(%)	2541 (9.1%)	52 (7.9%)	227 (8.5%)	547 (9.3%)	882 (9.2%)	833 (9.0%)	0.505
GCS at arrival	Median (IQR)	14 (10–15)	13 (7–15)	13 (8–15)	14 (9–15)	14 (10–15)	14 (11–15)	< 0.001
	Mild, n(%)	15,582 (55.6%)	284 (43.2%)	1263 (47.3%)	3163 (53.8%)	5449 (56.9%)	5423 (58.7%)	< 0.001
	Moderate, n(%)	6316 (22.5%)	167 (25.4%)	697 (26.1%)	1379 (23.5%)	2063 (21.6%)	2010 (21.8%)	< 0.001
	Severe, n(%)	6117 (21.8%)	206 (31.4%)	708 (26.5%)	1336 (22.7%)	2060 (21.5%)	1807 (19.6%)	< 0.001
Systolic BP^a^	Median (IQR), mmHg	156 (135–177)	160 (138–180)	157.5 (136–180)	156 (135–177)	155 (134–176)	157 (137–178)	< 0.001
	Hypotension on arrival, n(%)	647 (2.3%)	24 (3.7%)	86 (3.2%)	138 (2.3%)	219 (2.3%)	180 (1.9%)	0.930
Anticoagulant/platelet	n(%)	1379 (4.9%)	9 (1.4%)	62 (2.3%)	207 (3.5%)	457 (4.8%)	644 (7.0%)	< 0.001
Major comorbidity	n(%)	8019 (28.6%)	295 (44.9%)	928 (34.8%)	1820 (31.0%)	2666 (27.9%)	2310 (25.0%)	< 0.001
Max head AIS	3, n(%)	8419 (30.1%)	176 (26.8%)	797 (29.9%)	1708 (29.1%)	2870 (30.0%)	2868 (31.0%)	0.006
	4, n(%)	12,708 (45.4%)	255 (38.8%)	1125 (42.2%)	2679 (45.6%)	4396 (45.9%)	4253 (46.0%)	< 0.001
	5, n(%)	6888 (24.6%)	226 (34.4%)	746 (28.0%)	1491 (25.4%)	2306 (24.1%)	2119 (22.9%)	< 0.001
ISS	Median (IQR)	16 (13–24)	17 (14–25)	16 (13–25)	16 (14–25)	16 (13–24)	16 (13–24)	< 0.001
Operation for TBI	n(%)	4934 (17.6%)	164 (25.0%)	550 (20.6%)	1131 (19.2%)	1705 (17.8%)	1384 (15.0%)	< 0.001

^a^including missing data (*n* = 169)

*TBI* Traumatic brain injury, *GCS* Glasgow Coma Scale, *BP* Blood pressure, *AIS* Abbreviated Injury Scale, *ISS* Injury Severity Score, *IQR* Interquartile range

**Table 2:** Baseline characteristics of severe multiple trauma patients by year

		Total	2004–2006	2007–2009	2010–2012	2013–2015	2016–2018	p for trend
		*N* = 9978	*N* = 248	*N* = 1050	*N* = 2152	*N* = 3356	*N* = 3172	
Age	Median (IQR), years	76 (70–82)	73 (69–79.5)	74.5 (70–80)	76 (70–81)	76 (71–82)	77 (71–83)	< 0.001
	65–74	4311 (43.2%)	134 (54.0%)	525 (50.0%)	942 (43.8%)	1444 (43.0%)	1266 (39.9%)	< 0.001
	75–84	4077 (40.9%)	95 (38.3%)	398 (37.9%)	876 (40.7%)	1366 (40.7%)	1342 (42.3%)	0.013
	85-	1590 (15.9%)	19 (7.7%)	127 (12.1%)	334 (15.5%)	546 (16.3%)	564 (17.8%)	< 0.001
Sex	Male, n(%)	6056 (60.7%)	156 (62.9%)	646 (61.5%)	1281 (59.5%)	2051 (61.1%)	1922 (60.6%)	0.819
Type of trauma	Blunt, n(%)	9837 (98.6%)	240 (96.8%)	1035 (98.6%)	2124 (98.7%)	3309 (98.6%)	3129 (98.6%)	0.295
Mechanism of trauma	Traffic accident, n(%)	5544 (55.6%)	174 (70.2%)	653 (62.2%)	1269 (59.0%)	1854 (55.2%)	1594 (50.3%)	< 0.001
	Fall, n(%)	3887 (39.0%)	57 (23.0%)	330 (31.4%)	769 (35.7%)	1324 (39.5%)	1407 (44.4%)	< 0.001
	Others, n(%)	547 (5.5%)	17 (6.9%)	67 (6.4%)	114 (5.3%)	178 (5.3%)	171 (5.4%)	0.250
Cause of trauma	Accident, n(%)	9100 (91.2%)	232 (93.5%)	953 (90.8%)	1966 (91.4%)	3065 (91.3%)	2884 (90.9%)	0.498
Transfer system	Ambulance, n(%)	7572 (75.9%)	212 (85.5%)	841 (80.1%)	1655 (76.9%)	2523 (75.2%)	2341 (73.8%)	< 0.001
	Physician staffed ambulance/helicopter, n(%)	2140 (21.4%)	26 (10.5%)	165 (15.7%)	446 (20.7%)	746 (22.2%)	757 (23.9%)	< 0.001
	Others, n(%)	266 (2.7%)	10 (4.0%)	44 (4.2%)	51 (2.4%)	87 (2.6%)	74 (2.3%)	0.007
GCS at arrival	Median (IQR)	13 (7–14)	9.5 (4–14)	12 (6–14)	13 (7–14)	13 (7–14)	13 (7–15)	< 0.001
	Mild, n(%)	4328 (43.4%)	77 (31.0%)	404 (38.5%)	873 (40.6%)	1488 (44.3%)	1486 (46.8%)	< 0.001
	Moderate, n(%)	2509 (25.1%)	55 (22.2%)	254 (24.2%)	565 (26.3%)	835 (24.9%)	800 (25.2%)	0.656
	Severe, n(%)	3141 (31.5%)	116 (46.8%)	392 (37.3%)	714 (33.2%)	1033 (30.8%)	886 (27.9%)	< 0.001
Systolic BP^a^	Median (IQR), mmHg	134 (106–160)	123 (97–154)	129 (100–156)	132 (102–158)	134 (107–160)	136 (110–162)	< 0.001
	Hypotension on arrival, n(%)	1583 (15.9%)	52 (21.0%)	216 (20.6%)	378 (17.6%)	508 (15.1%)	429 (13.5%)	< 0.001
Anticoagulant/platelet	n(%)	289 (2.9%)	4 (1.6%)	16 (1.5%)	48 (2.2%)	91 (2.7%)	130 (4.1%)	< 0.001
Major comorbidity	n(%)	4001 (40.1%)	139 (56.0%)	503 (47.9%)	935 (43.4%)	1329 (39.6%)	1095 (34.5%)	< 0.001
Max head AIS	3, n(%)	3770 (37.8%)	92 (37.1%)	401 (38.2%)	792 (36.8%)	1303 (38.8%)	1182 (37.3%)	0.982
	4, n(%)	3813 (38.2%)	67 (27.0%)	370 (35.2%)	824 (38.3%)	1293 (38.5%)	1259 (39.7%)	< 0.001
	5, n(%)	2395 (24.0%)	89 (35.9%)	279 (26.6%)	536 (24.9%)	760 (22.6%)	731 (23.0%)	< 0.001
ISS	Median (IQR)	29 (25–38)	34 (25–41)	32 (25–41)	32 (25–41)	29 (25–38)	29 (25–38)	< 0.001
Operation for TBI	n(%)	1207 (12.1%)	38 (15.3%)	142 (13.5%)	274 (12.7%)	399 (11.9%)	354 (11.2%)	0.005
Surgical intervention for other region injury	n(%)	9932 (99.5%)	248 (100.0%)	1046 (99.6%)	2142 (99.5%)	3342 (99.6%)	3154 (99.4%)	0.246

^a^including missing data (*n* = 110)

*TBI* Traumatic brain injury, *GCS* Glasgow Coma Scale, *BP* Blood pressure, *AIS* Abbreviated Injury Scale, *ISS* Injury Severity Score, *IQR* Interquartile range

Blunt trauma was the most common type of injury in both groups. The proportion of fall cases increased over time in both groups; older patients fell more often in the isolated TBI group than in the multiple trauma group. In both groups, trauma was mostly due to accidents. Most patients were transferred to hospitals by ambulance; the proportion of cases transported by ambulance/helicopter with a physician increased over time by approximately 2-fold in the isolated TBI and multiple trauma groups.

The median GCS score and systolic blood pressure at the time of admission to the ED were lower in the multiple trauma group than in the isolated TBI group, and the proportion of severe GCS cases or hypotension showed a decreasing trend in both groups over time. The proportion of patients with a medical history of anticoagulant/antiplatelet use increased over time. The most common maximum head AIS score in the isolated TBI group over time was 4, whereas that in the multiple trauma group was increasing from 3 to 4. Median ISS values were higher in the multiple trauma group than in the isolated TBI group. A decrease in the ISS of both groups was seen over time.

Tables 3 and 4 show the annual trends of the primary and secondary outcomes for isolated TBI and multiple trauma, respectively. The overall proportions of patients with in-hospital death were 16.38 and 26.7% in the isolated TBI and multiple trauma groups, respectively. The proportions of patients who died in the hospital were 29.5% during 2004–2006 and 14.2% during 2016–2018 in the isolated TBI group and 48.0% during 2004–2006 and 21.7% during 2016–2018 in the multiple trauma group. In both univariable and multivariable analyses, the OR decreased after adjusting for potential confounders. The adjusted ORs for a 3-year increment were 0.84 (95% CI: 0.81–0.88) and 0.78 (95% CI: 0.75–0.83) in the isolated TBI and multiple trauma groups, respectively.

**Table 3:** Primary and secondary outcomes by year

	Total	2004–2006	2007–2009	2010–2012	2013–2015	2016–2018	
Isolated TB	*N* = 28,015	*N* = 657	*N* = 2668	*N* = 5878	*N* = 9572	*N* = 9240	
Death at hospital discharge	4588 (16.38%)	194 (29.5%)	560 (21.0%)	1058 (18.0%)	1467 (15.3%)	1309 (14.2%)	
							OR for 3–year increment
Crude OR		reference	0.63	0.52	0.43	0.39	0.83
95% CI			(0.52–0.77)	(0.44–0.63)	(0.36–0.52)	0.33–0.47)	(0.81–0.86)
Adjusted OR*		reference	0.63	0.57	0.45	0.42	0.84
95% CI			(0.49–0.81)	(0.45–0.72)	(0.35–0.57)	(0.33–0.53)	(0.81–0.88)
Death at emergency department	196 (0.70%)	7 (1.1%)	23 (0.9%)	50 (0.9%)	56 (0.6%)	60 (0.6%)	
							OR for 3–year increment
Crude OR		reference	0.81	0.80	0.55	0.61	0.88
95% CI			(0.34–1.89)	(0.36–1.76)	(0.25–1.20)	(0.28–1.33)	(0.77–1.00)
Adjusted OR**		reference	0.94	1.18	0.80	0.99	0.97
95% CI			(0.39–2.26)	(0.52–2.69)	(0.35–1.82)	(0.44–2.24)	(0.85–1.10)
	Total	2004–2006	2007–2009	2010–2012	2013–2015	2016–2018	
Multiple trauma	*N* = 9978	*N* = 248	*N* = 1050	*N* = 2152	*N* = 3356	*N* = 3172	
Death at hospital discharge	2668 (26.7%)	119 (48.0%)	352 (33.5%)	686 (31.9%)	822 (24.5%)	689 (21.7%)	
							OR for 3–year increment
Crude OR		reference	0.55	0.51	0.35	0.3	0.78
95% CI			(0.41–0.72)	(0.39–0.66)	(0.27–0.46)	(0.23–0.39)	(0.75–0.81)
Adjusted OR*		reference	0.56	0.57	0.37	0.32	0.78
95% CI			(0.40–0.80)	(0.41–0.79)	(0.27–0.51)	(0.23–0.45)	(0.75–0.83)
Death at emergency department	395 (3.96%)	18 (7.3%)	54 (5.1%)	105 (4.9%)	120 (3.6%)	98 (3.1%)	
							OR for 3–year increment
Crude OR		reference	0.69	0.66	0.47	0.41	0.81
95% CI			(0.40–1.20)	(0.39–1.10)	(0.28–0.79)	(0.24–0.69)	(0.74–0.89)
Adjusted OR**		reference	0.80	0.83	0.66	0.65	0.90
95% CI			(0.44–1.45)	(0.47–1.45)	(0.38–1.15)	(0.37–1.15)	(0.82–1.00)

*OR* Odds ratio, *CI* Confidence Interval

**Table 4:** The rate of TBI operation by age group

	Total	65–74	75–84	85–	
Isolated TBI	*N* = 28,015	*N* = 10,294	*N* = 11,753	*N* = 5968	*p*-value
Burr hole surgery in the ED, n(%)	628 (2.2%)	288 (2.8%)	242 (2.1%)	98 (1.6%)	< 0.001
Evacuation of hematoma, n(%)	4230 (15.1%)	1694 (16.5%)	1750 (14.9%)	786 (13.2%)	< 0.001
Decompressive craniectomy, n(%)	909 (3.2%)	465 (4.5%)	348 (3.0%)	96 (1.6%)	< 0.001
	Total	65–74	75–84	85–	
Multiple trauma	*N* = 9978	*N* = 4311	*N* = 4077	*N* = 1590	*p*-value
Burr hole surgery in the ED, n(%)	286 (2.9%)	133 (3.1%)	126 (3.1%)	27 (1.7%)	0.01
Evacuation of hematoma, n(%)	855 (8.6%)	429 (10.0%)	348 (8.5%)	78 (4.9%)	< 0.001
Decompressive craniectomy, n(%)	297 (3.0%)	170 (3.9%)	116 (2.8%)	11 (0.7%)	< 0.001

Mortality associated with preventable trauma death measured by TRISS also decreased over time (Additional file 2). The factor with the strongest influence on the primary outcome was severe GCS on arrival for both the isolated TBI (adjusted OR = 18.17, 95% CI: 16.26–20.31, *p* < 0.001) and multiple trauma (adjusted OR = 9.30, 95% CI: 8.00–10.81, *p* < 0.001) groups, followed by maximum head AIS code of 5 for the isolated TBI group (adjusted OR = 8.87; 95% CI: 7.72–10.19, *p* < 0.001) and age 95–99 years for the multiple trauma group (adjusted OR = 4.46, 95% CI: 2.37–8.41, *p* < 0.001) (Additional files 3 and 4).

The secondary outcome did not follow the same trend as that followed by the primary outcome. In multivariable analyses, the OR only decreased in the multiple trauma group. The adjusted ORs for a 3-year increment were 0.97 (95% CI: 0.85–1.10) in the isolated TBI group and 0.90 (95% CI: 0.92–1.00) in the multiple trauma group (Table 3).

Table 4 shows a comparison of in-hospital treatment according to age group. The proportion of TBI operation for both the isolated TBI and multiple trauma groups decreased as the age group increased.

## Discussion

Using data from a multicenter, retrospective, observational data registry in Japan, we demonstrated temporal trends in baseline characteristics and mortality among geriatric patients with TBI in a super-aged society. In-hospital mortality in both the isolated TBI and multiple trauma groups, and ED mortality in the multiple trauma group, significantly improved during the study period. This study of a super-aged society over a 15-year period is one of the largest retrospective studies comparing baseline characteristics and mortality among patients with TBI. As a higher proportion of patients with TBI are elderly, our research provides useful information for reducing mortality in aging societies, which are rapidly increasing worldwide.

Japan is one of the fastest aging societies, and the proportion of people aged > 65 years is expected to increase to 30% by 2025. Our study shows that with the aging of society, the proportion of oldest-old (age ≥ 85) patients with TBI also increases. In the USA, the TBI-related hospitalization rate was the highest among adults aged ≥75 years, followed by those aged 65–74 years and 55–64 years [23]. Similarly, in Japan, we observed a shift in the highest peak towards the more elderly population (aged 80 years) (Additional file 1). Although multiple trauma has been considered less frequent in elderly patients, we found that over time the proportion of oldest-old patients with TBI that had multiple trauma increased. This finding is consistent with that of recent reports, which suggest that an increase in the number of older multiple trauma patients is because of the growth in older adult population [1–5]. In addition, we found falls to be a predominant cause of TBIs in older adults. This finding is also consistent with the results of previous reports from other aging societies [24, 25]. As falls are lower kinetic events, an increase in falls is predicted to be associated with better outcomes, especially in elderly TBI patients.

Age is often correlated with poor clinical outcomes after isolated TBI and multiple trauma, as previously reported [6–8]. Several factors, including preexisting comorbidities, TBI-related systemic complications, increasing intracranial hematomas due to anticoagulants, and withdrawal of care in case of medical futility, may contribute to worse clinical outcomes in elderly people [26, 27]. An excessive progression of secondary brain injury, including brain swelling, probably contributes to the high mortality in older patients with an aging brain, whereas younger patients may still be able to overcome this damage [23]. Hence, we expected that mortality would increase as the society ages; however, in this study, over time, an improvement in clinical outcomes was observed after adjusting for various factors considered influencing the outcome [17–21]. This could be because some factors affecting clinical outcomes, such as improved prehospital activity, sophisticated team medical care, advanced ICU equipment, and the prevalence of trauma training courses, were not adjusted.

The odds ratio of the 3-year increment between isolated TBI and multiple trauma were not very different, but the mortality associated with multiple trauma, especially in-hospital mortality, was still higher than that for isolated TBI. Death due to trauma can be classified into the following periods: an immediate or almost immediate death in the field; death occurring 2–3 h after the injury due to respiratory distress or bleeding; and death occurring several days to 2–3 weeks later due to injury, multiorgan failure, or sepsis.

The first peak in traumatic deaths can be partially addressed by primary prevention measures. To reduce traumatic deaths caused by motor vehicle collisions, the Japanese Road Traffic Act was revised in June 2002 and imposes severe fines for traffic offenses; fatal collision caused by drunk drivers, for example, have decreased since then. Falls are a common cause of TBI in older adults, which may explain the high incidence of multiple trauma caused by lower energy injury in older adults compared with that caused by motor-accidents. These factors might be associated with the fact that the proportion of serious cases, reflected by a lower GCS score, hypotension, and higher ISS on arrival, decreased over time [17–21].

Furthermore, the second and third peaks could be influenced by medical intervention. The acute phase management of patients with severe TBI and multiple trauma presents a major challenge. Patients who arrived at JTDB-registered hospitals were managed according to the Japanese guidelines for managing patients with severe TBI [28]. These guidelines are similar to those by the Brain Trauma Foundation, USA [29]. Since the introduction of TBI guidelines, there has been a reduction in TBI-related hospital mortality rates [30], which could be the reason for improved clinical outcomes in this study. In brief, the mortality rate in multiple trauma patients has decreased in recent decades [31]. The Japan Advanced Trauma Evaluation and Care education program (equivalent to Advanced Trauma Life Support, USA) [32] was introduced in 2002 to avoid preventable trauma death. These education programs for better decision-making algorithms and treatment techniques may contribute to reduced mortality rates [33].

The mortality trend could also be affected by prehospital emergency medical services, such as improved ambulance systems and equipment [34]. We found that the mode of transfer of physician-staffed ambulance/helicopter had a conflicting effect on mortality for in the isolated TBI (adjusted OR = 0.90 95% CI: 0.79–1.03, *p* = 0.143) and multiple trauma (adjusted OR = 0.86 95% CI: 0.76–0.99, *p* = 0.031) groups (Additional files 3 and 4). A previous report using propensity score matching score showed that transfer by a helicopter with a physician led to reduced mortality in TBI patients than transfer by an ambulance [35]. Reduced rescue times and increased catchment areas would represent presumable specific advantages of helicopter. Furthermore, an ambulance or helicopter with a physician would be different from health care systems with other emergency medical services, in term of medical interventions performed by on-scene physicians; these advantages are considered to contribute to improved TBI mortality, especially in multiple trauma cases. Although we could not show that the mode of transport correlates with survival benefit in our study, which assessed trends in isolated TBI outcomes, this result should be confirmed in propensity score matching cohorts or randomized trials.

Improvement in mortality rates of elderly people who accounted for the largest subgroup in an aging society would lead to an improvement in overall mortality. In general, geriatric trauma patients had a higher incidence of infectious and thromboembolic complications than younger patients [36]. Adding to systematic complications because of limited intensive treatment resources, not many experts indicate craniotomy in geriatric patients with TBI [4]. However, the indication for surgery was not totally withdrawn, even in the oldest-old group, in our data (Table 4). In guidelines regarding elderly TBI patients, activities of daily living before the injury rather than age are used to determine surgical indications [28]. Indeed, neurosurgical interventions for elderly TBI patients in Japan are associated with improved neurological outcomes and reduced mortality [37]. Overall, our results may suggest that age should not be considered the sole contraindicating factor for surgery or intensive care in elderly patients. The next goal would be to identify which older adult subgroups would be better candidates for more aggressive treatments, such as surgery or ICU treatment, by analyzing not only survival outcomes but also neurological outcomes.

In the future, TBI in the older adult population may be expected to continue to increase. To prevent older adults from sustaining a TBI, it is important to implement measures to reduce falls and to educate people on treatment and outcomes. Creating step-free access at homes or remote monitoring of elderly people with disabilities using information and communication technology would be helpful as primary prevention measures for a fall. To minimize the adverse outcomes after TBI, it is also essential to educate not only older adults and their family members but also medical staff to know the characteristics and outcomes of TBI induced by a fall; for example, geriatric patients who were given antithrombotic drugs had a high risk for late exacerbation even in a fall [38].

### Limitations

This study had some limitations. First, there could be other unmeasured confounding factors that influenced outcomes. Second, the study primarily included blunt trauma cases; therefore, the results cannot be extended to penetrating TBI. For example, the second most common cause of TBIs in the USA is gunshot wounds, which are virtually unknown in Japan. Third, most patients arriving in JTDB-registered hospitals are in a serious condition. Patients with cardiac arrest upon hospital arrival and AIS codes of 6 or 9 were not included in the study, which could have led to a selection bias. Furthermore, the assessments of neurological outcomes such as the Glasgow outcome scale at 6 months to 1 year after trauma as well as Marshall computer tomography scale were unavailable in JTDB. TBI is the most common cause of death from trauma as well as acquired disability [1–3], and trends in the incidence of neurological outcomes after TBI are also needed in future research. Finally, as with all epidemiological studies, data integrity, validity, and ascertainment bias could be potential limitations. However, the use of a uniform data collection form provided by the JTDB for reporting trauma, a large sample size, and a multicenter-based design to cover a large extent of Japan was intended to minimize these potential sources of bias.

## Conclusion

Using the national JTDB registry, we demonstrated the number of elderly patients with TBI increased during the study period, and a nationwide reduction in TBI-related mortality despite the impact of age on cases of isolated TBI and multiple trauma. Our findings in the super-aged society of Japan could provide helpful clues for the treatment of TBI in patients worldwide.

## Supplementary Information

**Additional file 1.** Histogram of traumatic brain injury patients. TBI = traumatic brain injury.

**Additional file 2.** Trend for preventable trauma death.

**Additional file 3.** Factors associated with primary outcomes (isolated TBI).

**Additional file 4.** Factors associated with primary outcomes (multiple trauma).

## Data Availability

The data that support the findings of this study are available from the JTDB; restrictions apply to the availability of these data, which were used under license for the current study, and so are not publicly available. Data are, however, available from the authors upon reasonable request and with permission of the JTDB.

## Abbreviations

AIS: Abbreviated Injury Scale
CI: Confidence interval
ED: Emergency department
GCS: Glasgow Coma Scale score
ISS: Injury Severity Score
JTDB: Japanese Trauma Data Bank
OR: Odds ratio
TBI: Traumatic brain injury
TRISS: Trauma Injury Severity Score
