# Supplementary material for: Nationwide improvements in geriatric mortality due to traumatic brain injury in Japan
Source: BMC Emerg Med. 2022 Feb 10;22:24. doi: 10.1186/s12873-022-00577-w (PMC8830138; doi:10.1186/s12873-022-00577-w)
Supplement: Supplementary file 1 — Additional file 1. Histogram of traumatic brain injury patients. TBI = traumatic brain injury. [file 12873_2022_577_MOESM1_ESM.zip › HosomiSupplementalFigureLegends-3R3.docx]

**Figure Legends of Additional Files**

Additional file 1. Histogram of traumatic brain injury patients.

TBI = traumatic brain injury
