# Supplementary material for: Nationwide improvements in geriatric mortality due to traumatic brain injury in Japan
Source: BMC Emerg Med. 2022 Feb 10;22:24. doi: 10.1186/s12873-022-00577-w (PMC8830138; doi:10.1186/s12873-022-00577-w)
Supplement: Supplementary file 2 — Additional file 2. Trend for preventable trauma death. [file 12873_2022_577_MOESM2_ESM.docx]

| Additional file 2. Trend for preventable trauma death by year. | | | |  |  |  |
| --- | --- | --- | --- | --- | --- | --- |
|  | Total | 2004–2006 | 2007–2009 | 2010–2012 | 2013–2015 | 2016–2018 |
| Isolated TBI | N=28,015 | N=657 | N=2,668 | N=5,878 | N=9,572 | N=9,240 |
| Ps＜0.25 | 381/481 | 15/19 | 42/54 | 90/109 | 117/147 | 117/152 |
|  | 79.21% | 78.95% | 77.78% | 82.57% | 79.59% | 76.97% |
| 0.25≦Ps≦0.5 | 1345/2030 | 47/61 | 162/242 | 295/432 | 453/705 | 388/590 |
|  | 66.26% | 77.05% | 66.94% | 68.29% | 64.26% | 65.76% |
| Ps＞0.5 | 2244/21,879 | 94/463 | 282/2029 | 494/4337 | 701/7433 | 673/7617 |
|  | 10.26% | 20.30% | 13.90% | 11.39% | 9.43% | 8.84% |
|  |  |  |  |  |  |  |
|  | Total | 2004–2006 | 2007–2009 | 2010–2012 | 2013–2015 | 2016–2018 |
| Multiple trauma | N=9,978 | N=248 | N=1,050 | N=2,152 | N=3,356 | N=3,172 |
| Ps＜0.25 | 1230/1872 | 58/70 | 171/242 | 298/413 | 379/583 | 324/564 |
|  | 65.71% | 82.86% | 70.66% | 72.15% | 65.01% | 57.45% |
| 0.25≦Ps≦0.5 | 529/1271 | 25/44 | 74/142 | 133/299 | 173/436 | 124/350 |
|  | 41.62% | 56.82% | 52.11% | 44.48% | 39.68% | 35.43% |
| Ps＞0.5 | 589/5981 | 25/108 | 76/594 | 159/1206 | 163/2027 | 166/2046 |
|  | 9.85% | 23.15% | 12.79% | 13.18% | 8.04% | 8.11% |
|  |  |  |  |  |  |  |
| Ps：Probability of survival | |  |  |  |  |  |
