# Supplementary material for: Nationwide improvements in geriatric mortality due to traumatic brain injury in Japan
Source: BMC Emerg Med. 2022 Feb 10;22:24. doi: 10.1186/s12873-022-00577-w (PMC8830138; doi:10.1186/s12873-022-00577-w)
Supplement: Supplementary file 3 — Additional file 3. Factors associated with primary outcomes (isolated TBI). [file 12873_2022_577_MOESM3_ESM.docx]

| Additional file 3. Factors associated with primary outcomes (isolated TBI). | | | |  |  |  |  |  |  |
| --- | --- | --- | --- | --- | --- | --- | --- | --- | --- |
|  |  |  |  |  |  |  |  |  |  |
|  |  | All (N) | Death (N) | (%) | Crude OR | 95% CI | Adjusted OR | 95% CI | P value |
| Age | 65–69 | 4,921 | 679 | 13.80% | (reference) |  | (reference) |  |  |
|  | 70–74 | 5,373 | 767 | 14.28% | 1.04 | (0.93–1.16) | 1.12 | (0.98–1.28) | 0.103 |
|  | 75–79 | 5,931 | 924 | 15.58% | 1.15 | (1.04–1.28) | 1.36 | (1.19–1.56) | <0.001 |
|  | 80–85 | 5,822 | 1,034 | 17.76% | 1.35 | (1.21–1.50) | 1.81 | (1.59–2.07) | <0.001 |
|  | 85–89 | 3,957 | 781 | 19.74% | 1.54 | (1.37–1.72) | 2.27 | (1.96–2.63) | <0.001 |
|  | 90–94 | 1,621 | 322 | 19.86% | 1.55 | (1.34–1.79) | 2.15 | (1.78–2.60) | <0.001 |
|  | 95–99 | 340 | 74 | 21.76% | 1.74 | (1.33–2.28) | 2.87 | (2.02–4.06) | <0.001 |
|  | 100– | 50 | 7 | 14.00% | 1.02 | (0.46–2.27) | 2.01 | (0.79–5.12) | 0.141 |
| Sex | Female | 10,891 | 1,587 | 14.57% | (reference) |  | (reference) |  |  |
|  | Male | 17,124 | 3,001 | 17.53% | 1.25 | (1.17–1.33) | 1.31 | (1.21–1.43) | <0.001 |
| Type of trauma | Non-blunt | 904 | 212 | 23.45% | (reference) |  | (reference) |  |  |
|  | Blunt | 27,111 | 4,376 | 16.14% | 0.63 | (0.54–0.74) | 1.00 | (0.80–1.26) | 0.988 |
| Mechanism of trauma | Traffic accident | 7,107 | 1,232 | 17.34% | (reference) |  | (reference) |  |  |
|  | Fall | 18,582 | 2,830 | 15.23% | 0.86 | (0.80–0.92) | 0.93 | (0.85–1.03) | 0.158 |
|  | Others | 2,326 | 526 | 22.61% | 1.39 | (1.24–1.56) | 0.81 | (0.68–0.97) | 0.021 |
| Cause of trauma | Non-accident | 2,518 | 546 | 21.68% | (reference) |  | (reference) |  |  |
|  | Accident | 25,497 | 4,042 | 15.85% | 0.68 | (0.62–0.75) | 0.95 | (0.82–1.11) | 0.548 |
| Transfer system | Ambulance | 23,283 | 3,892 | 16.72% | (reference) |  | (reference) |  |  |
|  | Physician staffed ambulance/helicopter | 2,191 | 499 | 22.77% | 1.47 | (1.32–1.63) | 0.90 | (0.79–1.03) | 0.143 |
|  | Others | 2,541 | 197 | 7.75% | 0.42 | (0.36–0.49) | 0.79 | (0.66–0.94) | 0.009 |
| GCS at arrival | Mild | 15,582 | 531 | 3.41% | (reference) |  | (reference) |  |  |
|  | Moderate | 6,316 | 823 | 13.03% | 4.25 | (3.7–4.76) | 3.22 | (2.86–3.63) | <0.001 |
|  | Severe | 6,117 | 3,234 | 52.87% | 31.80 | (28.77–35.14) | 18.17 | (16.26–20.31) | <0.001 |
| Hypotension on arrival | No | 27,368 | 4,297 | 15.70% | (reference) |  | (reference) |  |  |
|  | Yes | 647 | 291 | 44.98% | 4.39 | (3.75–5.14) | 2.79 | (2.26–3.43) | <0.001 |
| Anticoagulant/platelet therapy | No | 26,636 | 4,307 | 16.17% | (reference) |  | (reference) |  |  |
|  | Yes | 1,379 | 281 | 20.38% | 1.33 | (1.16–1.52) | 1.43 | (1.20–1.71) | <0.001 |
| Major comorbidity | No | 19,996 | 2,927 | 14.64% | (reference) |  | (reference) |  |  |
|  | Yes | 8,019 | 1,661 | 20.71% | 1.52 | (1.43–1.63) | 1.22 | (1.12–1.33) | <0.001 |
| Max head AIS | 3 | 8,419 | 324 | 3.85% | (reference) |  | (reference) |  |  |
|  | 4 | 12,708 | 1,256 | 9.88% | 2.74 | (2.42–3.11) | 2.42 | (2.11–2.77) | <0.001 |
|  | 5 | 6,888 | 3,008 | 43.67% | 19.37 | (17.17–21.86) | 8.87 | (7.72–10.19) | <0.001 |
| Operation for TBI | No | 23,081 | 3,233 | 14.01% | (reference) |  | (reference) |  |  |
|  | Yes | 4,934 | 1,355 | 27.46% | 2.32 | (2.16–2.50) | 0.56 | (0.51–0.62) | <0.001 |
|  |  |  |  |  |  |  |  |  |  |
| TBI = traumatic brain injury, GCS = Glasgow Coma Scale, AIS = Abbreviated Injury Scale, ISS = Injury Severity Score | | | | | | |  |  |  |
