# Supplementary material for: Nationwide improvements in geriatric mortality due to traumatic brain injury in Japan
Source: BMC Emerg Med. 2022 Feb 10;22:24. doi: 10.1186/s12873-022-00577-w (PMC8830138; doi:10.1186/s12873-022-00577-w)
Supplement: Supplementary file 4 — Additional file 4. Factors associated with primary outcomes (multiple trauma). [file 12873_2022_577_MOESM4_ESM.docx]

| Additional file 4. Factors associated with primary outcomes (multiple trauma). | | | |  |  |  |  |  |  |
| --- | --- | --- | --- | --- | --- | --- | --- | --- | --- |
|  |  |  |  |  |  |  |  |  |  |
|  |  | All (N) | Death (N) | (%) | Crude OR | 95% CI | Adjusted OR | 95% CI | P value |
| Age | 65–69 | 2,132 | 427 | 20.03% | (reference) |  | (reference) |  |  |
|  | 70–74 | 2,179 | 526 | 24.14% | 1.27 | (1.10–1.47) | 1.35 | (1.14–1.62) | 0.001 |
|  | 75–79 | 2,215 | 637 | 28.76% | 1.61 | (1.40–1.85) | 1.93 | (1.62–2.29) | <0.001 |
|  | 80–85 | 1,862 | 570 | 30.61% | 1.76 | (1.52–2.04) | 2.31 | (1.93–2.77) | <0.001 |
|  | 85–89 | 1,117 | 343 | 30.71% | 1.77 | (1.50–2.09) | 3.02 | (2.45–3.71) | <0.001 |
|  | 90–94 | 396 | 141 | 35.61% | 2.21 | (1.75–2.78) | 4.49 | (3.36–6.00) | <0.001 |
|  | 95–99 | 69 | 22 | 31.88% | 1.87 | (1.11–3.14) | 4.46 | (2.37–8.41) | <0.001 |
|  | 100– | 8 | 2 | 25.00% | 1.33 | (0.27–6.62) | 4.90 | (0.82–29.36) | 0.082 |
| Sex | Female | 3,922 | 1,009 | 25.73% | (reference) |  | (reference) |  |  |
|  | Male | 6,056 | 1,659 | 27.39% | 1.09 | (0.99–1.19) | 1.22 | (1.08–1.37) | 0.001 |
| Type of trauma | Non-blunt | 141 | 39 | 27.66% | (reference) |  | (reference) |  |  |
|  | Blunt | 9,837 | 2,629 | 26.73% | 0.95 | (0.66–1.38) | 0.91 | (0.56–1.45) | 0.682 |
| Mechanism of trauma | Traffic accident | 5,544 | 1,675 | 30.21% | (reference) |  | (reference) |  |  |
|  | Fall | 3,887 | 836 | 21.51% | 0.63 | (0.58–0.70) | 1.04 | (0.91–1.18) | 0.583 |
|  | Others | 547 | 157 | 28.70% | 0.93 | (0.77–1.13) | 1.00 | (0.78–1.28) | 0.99 |
| Cause of trauma | Non-accident | 878 | 240 | 27.33% | (reference) |  | (reference) |  |  |
|  | Accident | 9,100 | 2,428 | 26.68% | 0.97 | (0.83–1.13) | 0.95 | (0.77–1.17) | 0.647 |
| Transfer system | Ambulance | 7,572 | 1,943 | 25.66% | (reference) |  | (reference) |  |  |
|  | Physician staffed ambulance/helicopter | 2,140 | 672 | 31.40% | 1.33 | (1.19–1.47) | 0.86 | (0.76–0.99) | 0.031 |
|  | Others | 266 | 53 | 19.92% | 0.72 | (0.53–0.98) | 0.84 | (0.58–1.24) | 0.386 |
| GCS at arrival | Mild | 4,328 | 320 | 7.39% | (reference) |  | (reference) |  |  |
|  | Moderate | 2,509 | 514 | 20.49% | 3.23 | (2.78–3.75) | 2.33 | (1.99–2.73) | <0.001 |
|  | Severe | 3,141 | 1,834 | 58.39% | 17.58 | (15.37–20.10) | 9.30 | (8.00–10.81) | <0.001 |
| Hypotension on arrival | No | 8,395 | 1,842 | 21.94% | (reference) |  | (reference) |  |  |
|  | Yes | 1,583 | 826 | 52.18% | 3.88 | (3.47–4.34) | 2.16 | (1.88–2.48) | <0.001 |
| Anticoagulant/platelet therapy | No | 9,689 | 2,608 | 26.92% | (reference) |  | (reference) |  |  |
|  | Yes | 289 | 60 | 20.76% | 0.71 | (0.53–0.95) | 1.09 | (0.77–1.54) | 0.641 |
| Major comorbidity | No | 5,977 | 1,236 | 20.68% | (reference) |  | (reference) |  |  |
|  | Yes | 4,001 | 1,432 | 35.79% | 2.14 | (1.95–2.34) | 1.45 | (1.29–1.62) | <0.001 |
| Max head AIS | 3 | 3,770 | 613 | 16.26% | (reference) |  | (reference) |  |  |
|  | 4 | 3,813 | 783 | 20.54% | 1.33 | (1.18–1.50) | 1.02 | (0.89–1.18) | 0.754 |
|  | 5 | 2,395 | 1,272 | 53.11% | 5.83 | (5.18–6.56) | 1.72 | (1.45–2.03) | <0.001 |
| Operation for TBI | No | 8,771 | 2,104 | 23.99% | (reference) |  | (reference) |  |  |
|  | Yes | 1,207 | 564 | 46.73% | 2.78 | (2.46–3.14) | 1.42 | (1.22–1.66) | <0.001 |
| Surgical intervention for other region injury | No | 46 | 33 | 71.74% | (reference) |  | (reference) |  |  |
|  | Yes | 9,932 | 2,635 | 26.53% | 0.14 | (0.07–0.27) | 0.45 | (0.22–0.92) | 0.028 |
| ISS |  | N/A | N/A |  | 1.08 | (1.08–1.09) | 1.04 | (1.03–1.05) | <0.001 |
|  |  |  |  |  |  |  |  |  |  |
| TBI = traumatic brain injury, GCS = Glasgow Coma Scale, AIS = Abbreviated Injury Scale, ISS = Injury Severity Score | | | | | | |  |  |  |
